# Substrate degradation and postbiotic analysis of alternative fiber ingredients fermented using an in vitro canine fecal inoculum model

**DOI:** 10.1093/jas/skad078

**Published:** 2023-03-21

**Authors:** Dalton A Holt, Isabella Corsato Alvarenga, Renan A Donadelli, Charles G Aldrich

**Affiliations:** Department of Grain Science and Industry, Kansas State University, Manhattan, KS 66506, USA; Department of Grain Science and Industry, Kansas State University, Manhattan, KS 66506, USA; Department of Grain Science and Industry, Kansas State University, Manhattan, KS 66506, USA; Department of Grain Science and Industry, Kansas State University, Manhattan, KS 66506, USA

**Keywords:** companion animals, fermentation, fruit, pomace, short-chain fatty acids, volatile fatty acids

## Abstract

Many fiber ingredients are used by the pet food industry; however, little data are available regarding the fermentation characteristics of alternative fibers currently being used. The objectives of this study were to determine organic matter disappearance (OMD) and postbiotic production from various fruit and vegetable fiber sources using an in vitro dog fecal inoculum model. Apple pomace (AP), blueberry pomace (BP), cranberry pomace (CP), tomato pomace (TP), and pea fiber (PF) were used as experimental treatments. Inoculum was prepared using freshly voided feces under anaerobic conditions. Predigested fibers were inoculated and incubated for 1, 3, 6, and 12 h at 39 °C. Short-chain fatty acids (SCFA), branched-chain fatty acids (BCFA), total volatile fatty acids (VFA), and OMD were determined for each fiber source and time point in triplicate. After 12 h of incubation, OMD was similar (P > 0.05; average of 18.5%) among treatments. Proportionally, acetate was greater for BP and AP (P < 0.05; average of 80.1%) than for the other treatments (68.3% to 71.2%). Molar proportions of propionate was greatest (P < 0.05) for CP (26.8%) compared to the remaining treatments (13.6 to 20.7%). Butyrate was proportionally greater for PF (7.7%; P < 0.05) than for BP and CP (average of 4.8%) and was lowest for AP (3.8%); however, TP was not different from PF (P > 0.05; average of 7.25%). Total VFA concentration was highest for AP (P < 0.05) followed by TP (1.17 and 0.75 mmol*g^−1^ of substrate, respectively). Both BP and PF were similar (average of 0.48 mmol*g^−1^ of substrate) and lower than for TP, with CP having the lowest VFA concentration (0.21 mmol*g^−1^ of substrate) among all treatments. Additionally, when comparing molar concentrations, AP and TP (average of 0.0476 mmol*g^−1^ of substrate) had greater butyrate concentrations than did PF (0.0344 mmol*g^−1^ of substrate). The AP, BP, and TP treatments had both linear and quadratic relationships (Table 7; P < 0.05) for acetate, propionate, and butyrate concentrations across time. CP only demonstrated a linear relationship for propionate (P < 0.05), whereas acetate and butyrate had quadratic relationships with time. PF only demonstrated quadratic relationships between acetate, propionate, and butyrate concentrations and time (P < 0.05). Overall, the fiber substrates evaluated were marginally to moderately fermentable when incubated for up to 12 h with canine fecal inoculum.

## Teaser

This work evaluated substrate degradation and postbiotic production of several alternative fiber ingredients that are currently used in the pet food industry. This data will provide valuable information to nutritionists and formulation scientists in the selection of cost-effective alternative fiber ingredients for the development of weight management and gut health diets.

## Introduction

Under the U.S. Food and Drug Administration’s Code of Federal Regulations, dietary fiber is defined as “nondigestible soluble and insoluble carbohydrates (with three or more monomeric units), and lignin that are intrinsic and intact in plants; isolated or synthetic nondigestible carbohydrates (with three or more monomeric units) determined by FDA to have physiological effects that are beneficial to human health” (FDA, 2022). Fiber is commonly included in pet food formulations due to the associated health benefits (Monti et al., 2016). Since dietary fiber represents carbohydrate substrates that escape digestion of the small intestine and arrive intact to the colon, incorporation of fiber into the diet is an effective means to lower caloric density. Dietary fibers are usually classified as either being insoluble or soluble (Williams et al., 2019). Solubility describes whether a fiber source will be dissolved by the digestive system secretions (soluble) or remain as discrete solid particles in the chyme (insoluble; Dikeman and Fahey, 2006). In general, it is accepted that greater solubility results in greater substrate degradation and fermentation by the microbiota; however, this is an oversimplification and is not necessarily predictive of behavior in the colon. Fibers that are fermented by the gut microbiota primarily result in the production of short-chain fatty acids (SCFA); these postbiotic compounds have a complex interaction between host, diet, and gut microbiome that appear to play an important role in long-term colonic health, metabolic control, and inflammatory status (Velázquez, 1996; Morrison and Preston, 2016). Fibers that resist degradation by the microbiota aid in stool formation and promote laxation.

For the last few decades, the use of fruit and vegetable byproducts in extruded foods has become a growing area of research (Karkle et al., 2012; Alavi et al., 2014). The residual biomass resulting from the processing of fruit and vegetables into consumer goods, termed pomace, is a major byproduct of the food industry (Ross et al., 2017). Further, the large quantity of these byproducts produced across the globe annually present challenges to prevent bioaccumulation in landfills. Pet food companies are constantly searching for novel ingredients to expand their portfolio and fruit and vegetable byproducts represent cost-effective, sustainable ingredient alternatives. With the exception of Swanson et al. (2001), data regarding fruit and vegetable fiber fermentation using a canine model is extremely limited. Thus, there is need to characterize the fermentation patterns of a wider array of fiber ingredients. It was hypothesized that fruit pomaces derived from apple, blueberry, and cranberry would contain the most fermentable fiber, tomato fiber would be intermediate, and PF would be the least fermentable. The objective of this study was to investigate substrate degradation and postbiotic production of several fibers that are currently used in pet diets, but have limited published data, using an in vitro canine inoculum model.

## Materials and Methods

### Fiber sources

A single source of apple pomace (AP), blueberry pomace (BP), cranberry pomace (CP), tomato pomace (TP), and pea fiber (PF) were obtained from a local mill (Lortscher Animal Nutrition, Inc., Bern, KS 66408) and used as experimental treatments. These fiber sources were selected based on their prevalence in diet formulations within the pet food industry but with limited reported data on potential benefit to gut health for companion animals. Prior to the in vitro protocols, fiber treatments were analyzed for dry matter (DM; AOAC 930.15), organic matter (AOAC 942.05), crude protein (AOAC 990.03), insoluble dietary fiber and total dietary fiber (TDF; AOAC 991.43; TDF kit, K-TDFR-200A, Megazyme Ltd., Bray, Ireland) according to AOAC Int’l approved analytical methodologies. Additionally, soluble fiber was calculated by the difference between TDF and insoluble fiber content. Crude protein of the residual samples remaining after the digestion procedure were also analyzed (AOAC 990.03) for the estimation of protein content entering fermentation.

### Media and dilution solution preparation

The medium composition and the anaerobic dilution solution were prepared according to Donadelli et al. (2019) and are detailed in Table 1. All components were autoclaved at 121 °C and 21 psi for 20 min with the exception of heat-liable vitamins that were filtered-sterilized and added to medium solution afterwards.

**Table 1.** Composition of the inoculum medium and anaerobic dilution solution

Component	Medium	Anaerobic dilution solution
Autoclaved		
Solution A^1^, mL	330.0	–
Solution B^2^, mL	330.0	–
Trace mineral solution^3^, mL	10.0	–
Resazurin solution^4^, mL	1.0	1.0
Yeast extract, g	0.5	–
Trypticase, g	0.5	–
Sodium carbonate monohydrate, g	4.0	–
Distilled water, mL	296.0	854.0
Cysteine hydrochloride monohydrate, g	0.5	0.5
Mineral solution #1^a^, mL	–	37.5
Mineral solution #2^b^, mL	–	37.5
Sodium bicarbonate solution^c^, mL	–	70.0
Added after autoclave		
Vitamin premix solution^5^, mL	20.0	–
Folate: biotin solution^6^, mL	5.0	–
Riboflavin solution^7^, mL	5.0	–
Hemin solution^8^, mL	2.5	–

^1^Solution A. 5.4 g sodium chloride, 5.4 g ammonium sulfate, 2.7 g potassium phosphate monobasic anhydrous, 0.18 g calcium chloride dihydrate, 0.12 g magnesium chloride hexahydrate, 0.06 g manganese chloride tetrahydrate, 0.06 g cobalt chloride hexahydrate. (Dilute to 1 Liter of distilled water).

^2^Solution B. 2.7 g potassium phosphate dibasic anhydrous. (Dilute to 1 Liter of distilled water.)

^3^Trace mineral solution. 500 mg of EDTA, 200 mg iron (II) sulfate heptahydrate, 30 mg m-phosphoric acid, 20 mg cobalt chloride hexahydrate, 10 mg zinc sulfate heptahydrate, 3 mg manganese chloride tetrahydrate, 3 mg sodium molybdate dihydrate, 2 mg nickel (II) chloride hexahydrate, 1 mg copper (II) chloride dihydrate. (Dilute to 1 Liter of distilled water.)

^4^Resazurin solution. 100 mg resazurin. (Dilute to 100 mL of distilled water.)

^5^Vitamin premix solution. 100 mg thiamin hydrochloride, 100 mg pantothenic acid, 100 mg niacin, 100 mg pyridoxine hydrochloride, 10 mg ammonium carbonate, 5 mg ρ-aminobenzoic acid, 0.25 mg vitamin B-12. (Dilute to 1 Liter of distilled water.)

^6^Folate: biotin solution − 100 mg ammonium carbonate, 10 mg folic acid, 2 mg biotin. (Dilute to 1 Liter of distilled water.)

^7^Riboflavin solution − 130 mg HEPES, 1 mg riboflavin. (Dilute to 1 Liter of distilled water.)

^8^Hemin Solution − 50 mg hemin, 40 mg sodium hydroxide. (Dilute to 100 mL of distilled water.)

^a^Mineral solution #1. 3 g potassium phosphate dibasic anhydrous, 1 g sodium citrate dehydrate. (Dilute to 500 mL of distilled water.)

^b^Mineral solution #2. 10 g sodium citrate dehydrate, 6 g sodium chloride, 6 g ammonium sulfate, 3 g potassium dihydrogen phosphate anhydrous, 1.23 g magnesium sulfate heptahydrate, 0.6 g calcium chloride dehydrate. (Dilute to 500 mL of distilled water.)

^c^Sodium bicarbonate solution, 91 g sodium bicarbonate. (Dilute to 1 Liter of distilled water.)

### Substrate preparation

To prepare treatments for inoculation, fiber sources underwent an in vitro digestion procedure using α-amylase, protease, and amyloglucosidase enzymes supplied from standard assay kit (TDF assay kit, Sigma-Aldrich, catalog no. TDF100A-1KT) to simulate small intestinal digestion adapted from the enzymatic TDF procedure described by Donadelli et al. (2019). Briefly, 10 g of sample was added with 500 mL 0.08 M phosphate buffer (pH 6) and a magnetic stirring bar and mixed until samples were dispersed into solution. The buffered sample mixtures were added with 1 mL of heat-stable α-amylase and incubated in a water bath at 95 °C for at least 15 min under continuous agitation to gelatinize and hydrolyze digestible starch content. After incubation, sample pH was increased to 7.5 with dilute sodium hydroxide (0.275 N) after reaching room temperature. Next, 1 mL of protease was added to each sample and incubated in a 60 °C water bath for at least 30 min under continuous agitation to solubilize and depolymerize protein. After the second incubation, sample pH was lowered to 4.3 with dilute hydrochloric acid (0.325 N) after reaching room temperature. Next, 1 mL of amyloglucosidase was added to each sample and incubated in a 60 °C water bath for at least 30 min under continuous agitation to further hydrolyze starch dextrins. After the final incubation, samples were added with 4 volumes of 95% ethanol to precipitate soluble fiber and allowed to sit overnight. Samples were filtered the following day and rinsed with two 100 mL volumes of 95% ethanol followed by two 100 mL volumes of acetone.

Residual samples remaining after enzymatic digestion were ground through a 1 mm screen in a fixed blade laboratory mill (Retsch, type ZM200, Haan, Germany). For each treatment, 300 ± 0.1 mg of predigested and ground substrate was placed into a 50-mL polypropylene centrifuge tube (Falcon brand conical centrifuge tubes; Corning Inc.) in triplicate. Additionally, three blank tubes were used during each time point for correction of background postbiotic concentrations present in fecal inoculum. Twenty-six milliliters of media solution were added to all tubes while purged with CO_2_ to maintain anaerobic conditions. Tubes were sealed with rubber stoppers equipped with one-way valves and allowed to hydrate overnight at 4 °C.

### Dog donors and inoculum preparation

Beagle dogs were used as donors for the preparation of fecal inoculum in this study. Dogs (*n* = 12, neutered/spayed, average age 5.4 ± 0.3 yr old, average weight 12.7 ± 1.5 kg) were group-housed in indoor kennels located at the Large Animal Research Center of Kansas State University in Manhattan, KS. Dogs were fed a commercial maintenance diet twice a day (Table 2) with constant access to fresh water. Fecal inoculum was prepared as described by Donadelli et al. (2019). Briefly, newly voided fecal samples were collected from two dogs (one male and one female) within 5 min of defecation, sealed in a sterile polyethylene bag (Whirl-Pak; Nasco sampling, Madison, WI), and stabilized at 39 °C until inoculum preparation. Fecal inoculum was produced immediately after collection by combining 50 g of sample from each donor and diluting 1:10 (w/v) with anaerobic dilution solution (100 g of feces: 900 mL of dilution solution). Inoculum was blended and filtered through four layers of cheese cloth under constant CO_2_ purge. The filtrate was retained in 1 L screw cap Pyrex bottle (Corning Inc., Corning, NY) at 39 °C and was immediately inoculated into tubes to begin incubation.

**Table 2.** Nutrient composition (DM-basis) of diet fed to inoculum donors

Nutrients	Diet composition
Crude protein, %	27.5
Fat (acid-hydrolyzed), %	17.8
Fiber (crude), %	2.5
Neutral detergent fiber, %	8.3
Acid detergent fiber, %	3.0
Nitrogen free extract (difference), %	37.4
Gross energy, kcal/kg	4,710
Digestible energy, kcal/kg	3,900
Ash, %	6.6

Ingredients: poultry byproduct meal, ground yellow corn, ground rice, corn gluten meal, poultry fat preserved with ethoxyquin, meat meal, ground wheat, beet pulp, animal digest, brewers dried yeast, animal fat preserved with BHA, dried whole egg, dried whey, soybean oil, calcium carbonate, L-lysine, potassium chloride, salt, pyridoxine hydrochloride, choline chloride, DL-methionine, menadione dimethylpyrimidinol bisulfate (source of vitamin K), cholecalciferol, biotin, lecithin, vitamin A acetate, DL-alpha tocopheryl acetate, inositol, ethoxyquin (a preservative), sodium selenite, calcium pantothenate, thiamin mononitrate, riboflavin, nicotinic acid, cyanocobalamin, L-tryptophan, folic acid, ferrous sulfate, manganous oxide, zinc oxide, copper sulfate, calcium iodate, cobalt carbonate.

### Fiber incubation and OMD determination

Prior to fecal collection, tubes containing treatments and media were placed in a water bath at 39 °C for at least 1 h to equilibrate temperature to 39 °C prior to inoculation. All treatments were incubated at four timepoints: 1, 3, 6, and 12 h. Tubes were inoculated with 4 mL of fecal inoculum starting from the longest time point (12 h) and ending with the earliest time point (1 h). During inoculation, tubes remained under CO_2_ purge until resealed with one-way valves. After each incubation time, duplicate 1 mL aliquots of clear supernatant were collected, acidified with 0.25 mL of 25% m-phosphoric acid, centrifuged at 25,000 × *g* for 20 min, and frozen at –20 °C. The remaining sample contents of each tube were transferred to a beaker and precipitated by diluting with 112 mL of 95% ethanol overnight. The precipitated samples were later filtered under vacuum using ashless filter paper (Whatman 541 ashless paper filter; catalog no. 1541-110) and 78% ethanol. When all sample volume had been filtered, residue was rinsed with two 10 mL volumes of 95% ethanol followed by two 10 mL volumes of acetone. After filtration, residues and filter paper were dried in a convection oven overnight at 105 °C (AOAC 930.15). Dry residue weight was recorded the following day and placed in muffle furnace at 450 °C overnight for determination of organic material (AOAC 942.05). Organic matter disappearance (OMD) was calculated using the following equation:


$$\begin{array}{l} OMD\;(\%)\;=\left[1\;-\;\left(\frac{OM\;residue\;-\;OM\;blank\;}{Initial\;OM}\right)\right]\;\times 100, \end{array}$$

where OM residue is the organic matter remaining in sample after fermentation and filtration in g, OM blank is the organic matter remaining in blank after fermentation and filtration in g, and initial OM is the organic matter of the raw sample in g.

### Postbiotics determination

The duplicate supernatant subsamples removed from each tube at the end of each time point were used for postbiotic analysis. Supernatants were extracted from gas chromatography vials and placed into a gas chromatograph (7890A GC System, Agilent Technologies, Santa Clara, CA) via direct liquid injection using a 10:1 split ratio with injection volume of 1 µL. The gas chromatography system used helium as a carrier gas with a flow rate of 3.5 mL/min and the volatile organic compounds were separated using a capillary gas chromatography column (15 m × 0.35 mm internal diameter, 0.5 µm film thickness; Nuko column, Sulpeco, Bellefonte, PA). A flame ionization detector was used for the determination of volatile compounds and was configured with nitrogen as the makeup gas with a flow rate of 25 mL/min to clarify peak resolution. Peak area of chromatograms was determined using integrative software (Agilent OpenLAB CDS version A.01.04, Agilent Technologies). Short-chain fatty acids (acetate, propionate, butyrate, and valerate) and branched-chain fatty acids (BCFA; isobutyrate and isovalerate) were quantified by comparing the sample peak area to a known standard of 10 mM concentration (Volatile Free Acid Mix, Sigma-Aldrich, St. Louis, MO). The mean value of the duplicate subsamples was used as concentration for each tube and concentrations were reported as mM^–1^ g of substrate on a DM-basis. For the purpose of this work, acetate, propionate, butyrate, and valerate were collectively referred to as SCFA. The volatile iso-acids, isobutyrate and isovalerate, were referred to as BCFA. Total volatile fatty acid (VFA) refers to the sum of them all.

### Statistical analysis

Data were analyzed as a completely randomized design with triplicate centrifuge tubes as experimental units (4 time points × 5 fiber sources × 3 replicates = 60 experimental units). Treatment least squares means within each timepoint for OMD and postbiotic compounds were compared using the general linear model procedure from statistical analysis software (SAS; V. 9.4, Cary, NC, USA). Pairwise treatment comparisons were conducted using Fisher’s Least Significance Difference to minimize type I error. Linear and quadratic trends across time within treatments were evaluated for concentrations of ­acetate, propionate, butyrate, and their sum using single degree of freedom orthogonal polynomial contrasts. ­Contrast co-efficients were estimated using the interactive matrix language procedure in SAS. For all statistical analyses, differences were considered significant at *P* < 0.05.

## Results

The nutrient composition of experimental fiber treatments is presented in Table 3. Treatment fibers had a large range in crude protein content (8.2% to 24.2%) with TP having the highest concentration, doubling that of the other treatments (average 11.9%). After digestion, TP still had the highest concentration of protein (21.4%) in residue samples, again roughly double that of the other treatments (average 10.8%). When evaluating TDF, TP was comparably lower (52.7%) than the other treatments (average 63.5%). CP had the highest TDF content (68.31%) but was almost completely insoluble (98.7% of TDF). As a proportion of TDF content, AP had a much greater amount of soluble fiber (17.2% of TDF) compared to the other treatments (1.3% to 7.6% of TDF)..

**Table 3.** Nutrient composition of treatment fiber sources

Composition, %	Fiber sources				
	Apple pomace	Blueberry pomace	Cranberry pomace	Pea fiber	Tomato pomace
Dry matter	91.3	91.4	94.8	90.9	91.9
	Dry matter basis				
Organic matter	97.9	98.7	98.0	97.2	95.9
Crude protein^1^	8.2 (9.4)	15.0 (13.8)	10.1 (11.0)	14.3 (9.1)	24.2 (21.4)
Total dietary fiber	63.4	62.8	68.3	59.5	52.7
Insoluble fiber^2^	52.5 (82.7)	58.9 (93.8)	67.4 (98.7)	55.0 (92.3)	50.0 (95.0)
Soluble fiber^3^	10.9 (17.2)	3.9 (6.2)	0.9 (1.3)	4.5 (7.6)	2.6 (5.0)

^1^Numbers in parenthesis represent crude protein concentration of residue after enzymatic digestion.

^2,^

^3^Numbers in parenthesis represent percentage of total fiber concentration.

After 12 h of incubation, there was no treatment effect (*P* > 0.05) on OMD (Table 5; average 18.5%). Acetate contributed the highest molar proportion of total VFA pool for each treatment across all time points (Table 5; 68.3% to 83.6%). After 1 h of incubation, all treatments had similar molar proportions of acetate (*P* > 0.05; average 78.4%); however, by the end of incubation (12 h), AP and BP had higher proportions of acetate (*P* < 0.05; average 80.1%) than the other treatments. Propionate was the second most abundant compound at all treatment and time point combinations and molar proportions differed (*P* < 0.05) among all treatments after 12 h of incubation. At the end of incubation, CP had the largest molar proportion of propionate (*P* < 0.05). Molar proportions of butyrate were smaller compared to acetate and propionate for each treatment and time point. After 12 h of incubation, PF had greater molar proportions of butyrate (*P* < 0.05; 7.7%) than AP, BP, and CP, but was similar (*P* > 0.05) to that of TP (average 7.2%).

**Table 5.** OMD^1^ and relative molar proportions of total VFA^2^ for individual and combined SCFA^3^ and BCFA^4^ of treatment fibers incubated with canine fecal inoculum at 1, 3, 6, and 12 h

Fermentation time, h	Fiber sources^5^					SEM	*P*-value
	AP	BP	CP	PF	TP		
OMD, %							
1	23.8^a^	15.7^a,b,c^	14.8^b,c^	11.3^c^	20.6^a,b^	1.78	0.0040
3	22.6^a^	12.0^b^	11.8^b^	8.2^b^	21.0^a^	2.05	0.0007
6	31.9^a^	17.3^b^	18.3^b^	14.7^b^	20.8^b^	2.52	0.0051
12	24.7	16.1	18.3	13.6	20.0	2.90	0.1605
Acetate, %							
1	78.9	79.5	78.1	75.80	79.5	0.93	0.0873
3	83.6^a^	81.7^b^	72.6^c^	73.1^c^	81.2^b^	0.34	<0.0001
6	80.9^a^	77.0^b^	69.1^d^	68.2^d^	73.8^c^	0.38	<0.0001
12	79.9^a^	80.3^a^	68.3^c^	70.4^b,c^	71.2^b^	0.61	<0.0001
Propionate, %							
1	18.4^a^	13.9^c^	16.9^b^	19.4^a^	16.5b	0.31	<0.0001
3	15.4^c^	14.2^c,d^	22.9^a^	19.6^b^	13.9^d^	0.32	<0.0001
6	16.1^c,d^	15.4^d^	25.1^a^	21.3^b^	16.5^c^	0.17	<0.0001
12	17.2^d^	13.6^e^	26.8^a^	20.7^b^	18.8^c^	0.28	<0.0001
Butyrate, %							
1	–0.3^b^	2.3^a^	3.7^a^	3.8^a^	2.8^a^	0.46	0.0005
3	2.2^c^	4.5^b^	4.2^b^	7.4^a^	4.5^b^	0.16	<0.0001
6	3.6^c^	5.6^b^	4.1^c^	7.5^a^	6.0^b^	0.21	<0.0001
12	3.8^c^	4.9^b,c^	4.7^b,c^	7.7^a^	6.8^a,b^	0.51	0.0015
Valerate, %							
1	2.8^a^	3.0^a^	0.0^b^	0.0^b^	0.0^b^	0.42	0.0004
3	0	0	0	0	0	0	–
6	0	0	0	0	0	0	–
12	0	0	0	0	0	0	–
SCFA, %							
1	99.8^a^	98.8^b,c^	98.7^c^	98.9^b^	98.9^b,c^	0.06	<0.0001
3	101.2^a^	100.4^a,b^	99.7^b^	100.1^b^	99.5^b^	0.19	0.0007
6	100.6^a^	98.1^b,c^	98.4^b^	96.9^c,d^	96.3^d^	0.27	<0.0001
12	100.9^a^	98.8^a,b^	99.8^a^	98.7^a,b^	96.8^b^	0.60	0.0084
Isobutyrate, %							
1	0	0	0	0	0	0	–
3	–0.3^b^	0.0^a,b^	0.1^a^	0.0^a,b^	0.3^a^	0.07	0.0034
6	0.0^c^	0.6^b^	0.7^b^	1.2^a^	1.4^a^	0.12	<0.0001
12	−0.1^a^	0.8^a,b^	0.4^a,b^	0.7^a,b^	1.4^a^	0.27	0.0386
Isovalerate, %							
1	0.2^c^	1.2^a,b^	1.3^a^	1.0^b^	1.1^a,b^	0.06	<0.0001
3	−1.0^b^	−0.4^a^	0.1^a^	−0.2^a^	0.2^a^	0.12	0.0004
6	−0.6^d^	1.3^b,c^	1.00^c^	1.8^a,b^	2.3^a^	0.16	<0.0001
12	−0.8^b^	0.4^a,b^	−0.2^b^	0.60^a,b^	1.8^a^	0.33	0.0027
BCFA, %							
1	0.2^c^	1.2^a,b^	1.3^a^	1.0^b^	1.1^a,b^	0.06	<0.0001
3	−1.2^e^	−0.4^d^	0.3^b,c^	−0.1^c,d^	0.5^a,b^	0.19	0.0007
6	−0.6^d^	1.9^b,c^	1.6^c^	3.1^a,b^	3.7^a^	0.27	<0.0001
12	−0.9^b^	1.2^a,b^	0.2^b^	1.2^a,b^	3.2^a^	0.60	0.0084

^1^OMD, organic matter disappearance.

^2^Total VFA, total volatile fatty acid (acetate + propionate + butyrate + valerate + isobutyrate + isovalerate).

^3^SCFA, short-chain fatty acid (acetate + propionate + butyrate + valerate).

^4^BCFA, branched-chain fatty acid (isobutyrate + isovalerate).

^5^AP, apple pomace; BP, blueberry pomace; CP, cranberry pomace; PF, pea fiber; TP, tomato pomace.

^a,b,c,d,e^Means within the same row with unlike superscripts differ.

Results for molar concentrations presented here will be exclusively focused on the 12 h time point. Total VFA concentrations were greatest (Table 6; *P* < 0.05) for AP (1.1647 mmol*g^–1^ of substrate) followed by TP (0.7530 mmol*g^–1^ of substrate), BP and CP (average 0.4804 mmol*g^–1^ of substrate), and lowest for PF (0.2049 mmol*g^–1^ of substrate). Butyrate concentrations were greater (*P* < 0.05) for AP and TP (average of 0.0476 mmol*g^–1^ of substrate), followed by PF (0.0344 mmol*g^–1^ of substrate), BP (0.0249 mmol*g^–1^ of substrate), and lowest for CP (0.0093 mmol*g^–1^ of substrate). Branched-chain fatty acid concentrations were greater for TP (*P* < 0.05; 0.0240 mmol*g^–1^) compared with the remaining treatments (ranging from –0.0101 to 0.0057 mmol*g^–1^). The AP, BP, and TP treatments had both linear and quadratic relationships (Table 7; *P* < 0.05) for acetate, propionate, and butyrate concentrations across time. CP only demonstrated a linear relationship for propionate (*P* < 0.05), whereas acetate and butyrate had quadratic relationships with time. PF only demonstrated quadratic relationships between acetate, propionate, and butyrate concentrations and time (*P* < 0.05).

**Table 6.** SCFA^1^, BCFA^2^, and total VFA^3^ concentrations of treatment fibers incubated with canine fecal inoculum at 1, 3, 6, and 12 h, expressed as mmol*g^−1^ of substrate (DM basis)

Fermentation time, h	Fiber sources^4^					SEM	*P*-value
	AP	BP	CP	PF	TP		
Acetate, mM*g^–1^							
1	0.1100^a,b^	0.0864^c^	0.0780^c^	0.0970^b,c^	0.1256^a^	0.00473	0.0002
3	0.5927^a^	0.1865^c^	0.1530^c^	0.1581^c^	0.3236^b^	0.01606	<0.0001
6	0.8130^a^	0.2723^c^	0.1194^d^	0.1907^c,d^	0.4278^b^	0.02266	<0.0001
12	0.9302^a^	0.4086^c^	0.1399^d^	0.3182^c^	0.5363^b^	0.02274	<0.0001
Propionate, mM*g^–1^							
1	0.0256^a^	0.0152^b^	0.0168^b^	0.0248^a^	0.0261^a^	0.00110	<0.0001
3	0.1094^a^	0.0325^c^	0.0478^b,c^	0.0423^b,c^	0.0554^b^	0.00360	<0.0001
6	0.1617^a^	0.0546^c^	0.0434^c^	0.0595^c^	0.0955^b^	0.00456	<0.0001
12	0.2005^a^	0.0695^d^	0.0546^d^	0.0933^c^	0.1417^b^	0.00476	<0.0001
Butyrate, mM*g^–1^							
1	–0.0005^b^	0.0025^a,b^	0.0037^a^	0.0048^a^	0.0045^a^	0.00070	0.0019
3	0.0158^a,b^	0.0103^b,c^	0.0088^c^	0.0161^a,b^	0.0178^a^	0.00141	0.0040
6	0.0358^a^	0.0198^b^	0.0072^c^	0.0212^b^	0.0349^a^	0.00180	<0.0001
12	0.0441^a^	0.0249^c^	0.0093d	0.0344^b^	0.0510^a^	0.00174	<0.0001
Valerate, mM*g^–1^							
1	0.0038^a^	0.0033^a^	0^b^	0^b^	0^b^	0.00051	0.0003
3	0	0	0	0	0	0	–
6	0	0	0	0	0	0	–
12	0	0	0	0	0	0	–
SCFA, mM*g^–1^							
1	0.1390^a,b^	0.1074^c,d^	0.0984^d^	0.1266^b,c^	0.1562^a^	0.0057	0.0002
3	0.7178^a^	0.2292^c^	0.2096^c^	0.2165^c^	0.3968^b^	0.0210	<0.0001
6	1.0104^a^	0.3467^c^	0.1700^d^	0.2713^c,d^	0.5582^b^	0.0287	<0.0001
12	1.1749^a^	0.5030^c^	0.2038^d^	0.4458^c^	0.7290^b^	0.0268	<0.0001
Isobutyrate, mM*g^–1^							
1	0	0	0	0	0	0	–
3	–0.0018^c^	0.0000^b^	0.0003^b^	0.0002^b^	0.0012^a^	0.00017	<0.0001
6	0.0005^c^	0.0022^b,c^	0.0012^c^	0.0035^b^	0.0082^a^	0.00038	<0.0001
12	–0.0008^d^	0.0040^b^	0.0012^c,d^	0.0030^b,c^	0.0103^a^	0.00059	<0.0001
Isovalerate, mM*g^–1^							
1	0.0003^b^	0.0013^a^	0.0013^a^	0.0013^a^	0.0018^a^	0.00013	0.0001
3	−0.0068^c^	−0.0008^b^	0.0003^a,b^	−0.0003^a,b^	0.0007^a^	0.00028	<0.0001
6	−0.0062^d^	0.0047^b^	0.0017^c^	0.0052^b^	0.0131^a^	0.00062	<0.0001
12	−0.0093^c^	0.0022^b^	0.0000^b^	0.0027^b^	0.0137^a^	0.00070	<0.0001
BCFA, mM*g^−1^							
1	0.0003^b^	0.0013^a^	0.0013^a^	0.0013^a^	0.0018^a^	0.00013	0.0001
3	−0.0087^c^	−0.0008^b^	0.0007^a,b^	−0.0002^b^	0.0018^a^	0.00043	<0.0001
6	−0.0057^d^	0.0068^b,c^	0.0028^c^	0.0087^b^	0.0213^a^	0.00098	<0.0001
12	−0.0101^c^	0.0062^b^	0.0012^b^	0.0057^b^	0.0240^a^	0.00126	<0.0001
Total VFA, mM*g^−1^							
1	0.1393^a,b^	0.1087^c,d^	0.0998^d^	0.1279^b,c^	0.1581^a^	0.00581	0.0002
3	0.7092^a^	0.2284^c^	0.2103^c^	0.2164^c^	0.3986^b^	0.02125	<0.0001
6	1.0048^a^	0.3535^c^	0.1728^d^	0.2800^c,d^	0.5795^b^	0.02921	<0.0001
12	1.1647^a^	0.5092^c^	0.2049^d^	0.4515^c^	0.7530^b^	0.02748	<0.0001

^1^SCFA, short-chain fatty acid (acetate + propionate + butyrate + valerate).

^2^BCFA, branched-chain fatty acid (isobutyrate + isovalerate).

^3^Total VFA, total volatile fatty acid (acetate + propionate + butyrate + valerate + isobutyrate + isovalerate).

^4^AP, apple pomace; BP, blueberry pomace; CP, cranberry pomace; PF, pea fiber; TP, tomato pomace.

^a,b,c,d,e^Means within the same row with unlike superscripts differ.

**Table 7.** Linear and quadratic relationships between concentrations (mmol*g^−1^ of substrate, DM basis) of acetate, propionate, butyrate, and their sum^1^ of treatment fibers over time

Fiber sources	Postbiotic, mM*g^−1^	Time points, h				Contrasts *P*-value	
		1	3	6	12	Linear	Quadratic
Apple pomace							
	Acetate	0.1100	0.5926	0.8130	0.9302	<0.0001	<0.0001
	Propionate	0.0256	0.1094	0.1617	0.2005	<0.0001	<0.0001
	Butyrate	−0.0005	0.0158	0.0358	0.0441	<0.0001	0.0019
	Sum	0.1351	0.7178	1.0104	1.1748	<0.0001	<0.0001
Blueberry pomace							
	Acetate	0.0864	0.1864	0.2723	0.4086	0.0043	0.0012
	Propionate	0.0152	0.0324	0.0546	0.0695	0.0004	0.0108
	Butyrate	0.0025	0.0103	0.0198	0.0249	0.0002	0.0114
	Sum	0.1040	0.2292	0.3467	0.5031	0.0022	0.0018
Cranberry pomace							
	Acetate	0.0780	0.1530	0.1194	0.1400	0.2414	0.0077
	Propionate	0.0168	0.0478	0.0434	0.0546	0.0285	0.0015
	Butyrate	0.0037	0.0088	0.0072	0.0093	0.1705	0.0034
	Sum	0.0984	0.2096	0.1700	0.2038	0.1474	0.0047
Pea fiber							
	Acetate	0.0970	0.1581	0.1907	0.3182	0.5212	0.0092
	Propionate	0.0248	0.0422	0.0595	0.0933	0.0760	0.0071
	Butyrate	0.0049	0.0161	0.0211	0.0344	0.0947	0.0101
	Sum	0.1266	0.2165	0.2713	0.4458	0.2938	0.0060
Tomato pomace							
	Acetate	0.1256	0.3236	0.4278	0.5363	<0.0001	<0.0001
	Propionate	0.0261	0.0554	0.0955	0.1417	<0.0001	<0.0001
	Butyrate	0.0045	0.0178	0.0349	0.0510	<0.0001	<0.0001
	Sum	0.1562	0.3967	0.5582	0.7290	<0.0001	<0.0001

^1^Sum: acetate + propionate + butyrate.

## Discussion

The nutrient composition of treatment fibers was evaluated to provide context to fermentation patterns (Table 3). In addition, previously reported values of fiber and protein content from prior studies evaluating these fiber sources are presented in Table 4 for comparison. Apple pomace has been well studied and its nutrient composition was within values reported throughout the literature (Lebet et al., 1998; Grigelmo-Miguel and Martin-Belloso, 1999; Swanson et al., 2001; Cerda-Tapia et al., 2015). Less work has been published on berry processing byproducts such as blueberry and cranberry pomace. The protein content of BP was greater than previously reported (6.6% to 9.0%; Crizel et al., 2016; Šarić et al., 2019; Tagliani et al., 2019; Hotchkiss et al, 2021) as was the TDF content which ranged widely within the available literature (26.2% to 56.4%; Crizel et al., 2016; Šarić et al., 2019; Tagliani et al., 2019; Hotchkiss et al., 2021). The protein content of CP was also greater than previously reported (2.2% to 8.2%; Park and Zhao, 2006; White et al., 2010; Spadoni Andreani and Karboune, 2020). Spadoni Andreani and Karboune (2020) reported TDF for cranberry pomace (63%), whereas White et al. (2010) reported insoluble dietary fiber (65.5%) which were reasonably similar to values reported for the current study. However, White et al. (2010) also reported a higher soluble dietary fiber (5.7%) content. Protein was greater for PF than that reported by Lebet et al. (1998) for pea hulls and Titgemeyer et al. (1991) for pea fiber (6.2% and 4.9%, respectively) but was similar to that reported by Swanson et al. (2001) for pea hull (16.2%). The TDF content of PF was lower than previously reported (69.7% to 89.7%; Titgemeyer et al., 1991; Lebet et al., 1998; Swanson et al., 2001; Donadelli et al., 2019). The protein content of TP was at the upper limits of previous reports (18.9% to 24.7%; Swanson et al., 2001; Savadkoohi and Farahnaky, 2012; Shao et al., 2013) but TDF was lower (Fahey et al., 1990; Swanson et al., 2001).

**Table 4.** Fiber and protein composition of fiber sources as reported by previous authors

Fiber treatment	Authors	Fiber, %			Protein, %
		TDF^1^	IDF^2^	SDF^3^	
Apple pomace					
	Cerda-Tapia et al. (2015)	70.9	59.8	11.1	3.9
	Grigelmo-Miguel and Martin-Belloso (1999)	60.1	46.3	13.8	5.1
	Lebet et al. (1998)	65.1	51.6	13.4	4.7
	Swanson et al. (2001)	79.3	68.1	11.2	6.4
Blueberry pomace					
	Crizel et al. (2016)	47.5	45.9	1.5	7.5
	Hotchkiss et al. (2021)	–	–	–	9.0
	Šarić et al. (2019)	56.4	54.7	1.7	–
	Tagliani et al. (2019)	26.1	–	–	6.6
Cranberry pomace					
	Park and Zhao (2006)	–	–	–	8.2
	Spadoni Andreani and Karboune (2020)	63.0	–	–	7.6
	White et al. (2010)	–	65.5	5.7	2.2
Pea fiber					
	Donadelli et al. (2019)	71.9	65.1	6.9	–
	Lebet et al. (1998) ^4^	87.5	81.3	6.2	6.2
	Swanson et al. (2001) ^4^	69.7	66.7	3.0	16.2
	Titgemeyer et al. (1991)	89.7	–	–	4.9
Tomato pomace					
	Fahey et al. (1990)	66.5	–	–	–
	Savadkoohi and Farahnaky (2012)	–	–	–	24.7
	Shao et al. (2013)	–	47.3	11.5	21.9
	Swanson et al. (2001)	56.9	52.7	4.2	18.9

–, values not reported.

^1^TDF, total dietary fiber.

^2^IDF, insoluble dietary fiber.

^3^SDF, soluble dietary fiber.

^4^Authors evaluated pea hulls.

Colonic transit times of beagle dogs has been reported between 12.2 and 14.8 h (De Cuyper et al., 2018). It is ­possible that the addition of various fiber sources could impact retention in the large bowel; however, previous results have been conflicting (Burrows et al., 1982; Fahey et al., 1990) and it is reasonable to assume that a transit time of at least 12 h is appropriate for modeling. In the present study, substrate degradation was quantified by the disappearance of organic matter after each incubation timepoint; however, after 12 h of incubation, there were no differences among treatments due to large variation within treatment replicates. Throughout incubation, the OMD values of TP remained relatively constant, similar to that reported previously by Swanson et al. (2001) for tomato pomace incubated between 0 and 12 h (24.4% to 26.5%, respectively); however, the values reported for this study were slightly lower. Due to the relative consistency in OMD values, Swanson et al. (2001) considered tomato pomace to be poorly fermented at 12 h and only after 24 h did these authors report an increase in OMD up to 35.0%. For the remaining treatment substrates evaluated here, OMD values across time were not consistent nor did they increase. Treatment substrates exhibited modest fermentation during 12 h of incubation in terms of postbiotic concentrations and likely only experienced a minor degree of substrate degradation during inoculation. Small fluctuations across time were likely error associated with fecal inoculum.

Among the postbiotic compounds produced from colonic fermentation, acetate, propionate, and butyrate usually comprise greater than 95% of molar concentrations (Bergman, 1990; den Besten et al., 2013). In most mammals, acetate is the most predominant SCFA and usually present in greater concentrations than all other postbiotics combined; however, butyrate is commonly recognized as the most important postbiotic compound produced during fermentation, particularity for the health of the colonic epithelium. Acetate is transported into circulation where it is taken up and metabolized as an energy source by many peripheral organs (den Besten et al., 2013), whereas propionate is primarily absorbed by the liver and serves as a precursor for gluconeogenesis (Miller and Wolin, 1996). Almost all of the butyrate formed in the colon is absorbed by the colonocytes where it is preferentially utilized as an energy source, serves as a regulator of cellular proliferation and differentiation, and has been shown to have beneficial effects on colonic disease (Velázquez et al., 1996; Alexander et al., 2019).

Apple fibers are known to contain significant portions of pectin (Guillon et al., 1995; Lebet et al., 1998) and several authors have previously reported that the fermentation of pectin structures greatly increases the production of acetate (Adiotomre et al., 1990; Titgemeyer et al., 1991; Barry et al., 1995). The relatively large portion (~17% of total fiber ­concentration) of soluble fiber of AP likely consisted mainly of pectic substances and would generally be expected to be susceptible to fermentation. Indeed, this was evident as the highest concentration of total VFA during incubation occurred with AP. The blueberry cell wall also consists of small proportions of soluble pectin as well as insoluble cellulose and hemicelluloses (Lin et al., 2019; Hotchkiss et al., 2021). While BP exhibited fermentation patterns characteristic of pectin substrates, such as an extremely high acetate:propionate ratio (Adiotomre et al., 1990; Titgemeyer et al., 1991; Sunvold et al., 1995a; Bourquin et al., 1996), the concentration of total VFA produced during incubation was very modest, which is consistent with the small fraction of soluble fiber. For the remaining treatment substrates, while still comprising the greatest contribution to the VFA pool, molar proportions of acetate decreased as incubation time progressed.

While the total VFA concentrations of CP appeared to plateau after only 3 h of incubation, propionate concentrations continued to increase. Indeed, among the three major SCFA (acetate, propionate, and butyrate), propionate was the only compound that demonstrated a linear relationship with time, whereas acetate, butyrate, and the sum of the three all demonstrated a quadratic relationship with time. Majeed et al. (2018) fermented cranberry seed fiber in vitro using isolated probiotic species *Bacillus coagulans* and found that after 6, 12, and 24 h of incubation, propionate concentrations far exceeded that of acetate and butyrate. While the concentrations of propionate did not exceed that of acetate and propionate for CP in the current study, the greater molar proportions of propionate, relative to other substrates, is likely a function of the unique polysaccharide structure of the cranberry cell wall. Previous reports have identified relatively abundant amounts of arabinose and galactose within its structure (Fan et al., 2010; Spadoni Andreani and Karboune, 2020; Spadoni Andreani et al., 2021). Human fermentation studies have also shown that the degradation of pentose sugars including arabinose and xylose lead to greater concentrations of propionate in vitro (Mortensen et al., 1988; Salvador et al., 1993).

Generally, PF had the largest proportion of butyrate during incubation; however, at 12 h pea fiber was not different from TP. Butyrate proportions were greater than that previously reported for PF (4.2%; Donadelli et al., 2019) and pea hulls (2.9%; Swanson et al., 2001); however, the molar concentration of butyrate was relatively similar (0.02824 and 0.0300 mmol*g^–1^ of substrate, respectively). While the molar proportion of butyrate from PF was higher compared to other sources, AP and TP produced the highest molar concentrations of butyrate. Pea fibers contain large amounts of insoluble cellulose (Guillon et al., 1995; Lebet el al., 1998), which is largely resistant to degradation in the colon of canines (Sunvold et al., 1995b; Swanson et al., 2001; Donadelli et al., 2019) and may explain the lower total VFA production.

Total VFA concentration of TP was about 65% that of AP with most of the difference represented by acetate. TP had similar concentrations of butyrate to that of AP, but proportionally butyrate made a greater contribution to the total VFA pool. Thus, TP contributed both greater concentrations and relative proportions of butyrate compared to other treatment fibers. However, TP also produced the largest concentration of BCFA. The presence of BFCA is a direct indicator of protein fermentation, whereby isobutyrate and isovalerate are formed by the metabolism of the branched-chain amino acids valine and leucine, respectively (Macfarlane et al., 1992). Protein fermentation results in a complex mixture of metabolites including SCFA, BCFA, ammonia, biogenic amines, phenolic, and indolic compounds to name a few. While only SCFA and BCFA were quantified, many of these other compounds are toxic to the colonocytes and have been associated with gut health impairment such as inflammation, reduced epithelial barrier function, and even tumor-promotion (Hughes et al., 2008; Gilbet et al., 2018; Trefflich et al., 2021).

Some interesting and useful points of data were observed during this study; however, there were a few limitations identified here. First, for each fiber treatment, only a single sample was sourced for substrate preparation. A composite sample composed from multiple sources of the same ingredient would have provided a better representation of each ingredient and helped account for batch-to-batch variability. However, treatments that have been previously evaluated demonstrated relative consistency in terms of nutrient composition as well as fermentation patterns. Second, while the postbiotic analysis showed good consistency among treatment replicates, the OMD values between replicates varied widely. Incomplete homogenization of fecal material during inoculum preparation was likely a source of error here, potentially masking any detectible differences in substrate degradation between treatment fibers. Additional replication would be necessary to draw inference on substrate degradation. Third, reference standards including a completely fermentable and a nonfermentable standard such as crystalline cellulose and isolated pectin, respectively, would have provided an upper and lower limit for treatment comparisons. However, PF and TP are commonly used within the pet food industry and have been previously evaluated using the same in vitro procedures. Thus, bridging previous reports to this current study. Finally, differences in fermentation patterns were likely a function of the unique monosaccharide composition in the cell walls of fiber treatments. Unfortunately, this was beyond the scope the current study. Future work should characterize the composition and disappearance of monosaccharides in these and other fibers to help elucidate the mechanisms driving differences in postbiotic production. Despite these limitations, this data provides valuable knowledge to the limited published research evaluating novel fiber ingredients in companion animal diets.

## Conclusion

The fruit and vegetable fibers evaluated here AP, BP, CP, PF, and TP were low to moderately fermentable with AP being the most fermentable and CP being the least. All fiber treatments had unique patterns of postbiotic production across time. However, there were no large shifts toward butyrate production demonstrated for any of the evaluated fiber sources. Potential remains for these alternative ingredients in companion animal diets to promote gastrointestinal health, weight management, and digestive regularity.

## Abbreviations

AP: apple pomace
BCFA: branched-chain fatty acid
BP: blueberry pomace
CP: cranberry pomace
DM: dry matter
OMD: organic matter disappearance
PF: pea fiber
SCFA: short-chain fatty acid
TDF: total dietary fiber
TP: tomato pomace
VFA: volatile fatty acid
